# Molecular Docking, Antitumor and Antimicrobial Activities of Extracts and Synthesized Silver Nanoparticles From *Piper chaba* Roots

**DOI:** 10.1002/fsn3.72087

**Published:** 2026-07-07

**Authors:** Sultana Rajia, Md. Ariful Islam, Md. Aminul Islam, Alima Khanam, Iffat Ara Tasnim, Md. Shamim Ahmed, Imtiaj Hasan

**Affiliations:** ^1^ Department of Nutrition and Food Engineering Varendra University Rajshahi Bangladesh; ^2^ Center for Interdisciplinary Research Varendra University Rajshahi Bangladesh; ^3^ Department of Genetic Engineering and Biotechnology University of Rajshahi Rajshahi Bangladesh; ^4^ Department of Biochemistry and Molecular Biology University of Rajshahi Rajshahi Bangladesh; ^5^ Department of Pharmacy Varendra University Rajshahi Bangladesh; ^6^ Department of Microbiology University of Rajshahi Rajshahi Bangladesh

**Keywords:** antimicrobial, antitumor, molecular docking, *Piper chaba*, silver nanoparticle

## Abstract

*Piper chaba*, a traditionally important spice and medicinal plant, was investigated for its anticancer and antimicrobial potential using crude root extracts and root‐derived synthesized silver/silver chloride nanoparticles. Cytotoxicity assessment using the brine shrimp nauplii assay revealed LC_50_ values of 2445.56, 1983.56, and 1306.98 μg/mL for the aqueous PcRE (Aq), ethanolic PcRE (Et) extracts, and nanoparticle formulations PcRE (Np), respectively, indicating enhanced bioactivity of the nanoparticles. Antibacterial evaluation showed that PcRE (Np) exhibited lower MBC/MIC ratios (1.60–2.64) compared to PcRE (Aq) (2.00–2.13) and PcRE (Et) (2.17–2.20), suggesting superior bactericidal efficacy. At 250 μg/mL, PcRE (Np) demonstrated the highest antibiofilm activity against *Escherichia coli* (ATCC 27853) (74%), followed by PcRE (Aq) (67%) and PcRE (Et) (42%). In vivo anticancer studies using Swiss albino mice bearing Ehrlich ascites carcinoma (EAC) showed tumor growth inhibition of 26.3%, 32.0%, and 41.5% for PcRE (Aq), PcRE (Et), and PcRE (Np), respectively, at a dose of 2.5 mg/kg/day. Consistently, MTT assays against EAC cells indicated higher growth inhibition by PcRE (Np) (12.7%) compared with PcRE (Et) (7.5%) and PcRE (Aq) (6.65%) at 300 μg/mL. Treatment with PcRE (Np) significantly prolonged survival and improved hematological parameters, while also modulating cancer‐associated blood glucose levels. Histopathological analysis revealed notable hepatic tissue recovery in PcRE (Np)‐treated groups, exceeding that observed with fluorouracil. Molecular docking studies further supported these findings, demonstrating strong interactions of major *P. chaba* phytochemicals with COX‐2 and p53 proteins. Collectively, these results highlight PcRE (Np) as a promising antimicrobial and anticancer candidate.

## Graphical abstract

This figure illustrates the preparation of extracts and synthesized silver nanoparticles from the roots of *Piper chaba*, a traditionally important spice and medicinal plant, along with their potential biological activities and interactions with p53 and COX‐2 proteins as revealed through molecular docking studies. All samples demonstrated significant antibiofilm and antitumor activities against Ehrlich ascites carcinoma cells in both in vitro and in vivo experiments. Supporting investigations, including evaluation of antidiabetic activity in mice, analysis of morphological alterations in cancer cells, histopathological examination of mouse liver tissues, and assessment of mouse survival rates, were also performed. In summary, these findings suggest that the two extracts and the synthesized silver nanoparticles represent promising candidates with antimicrobial and anticancer potential.

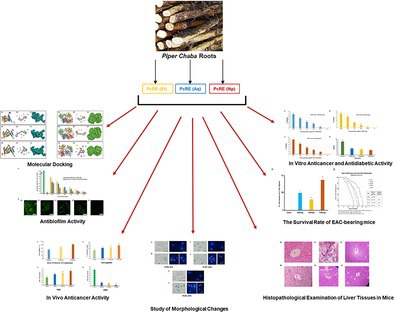

## 1 Introduction

Natural compounds derived from plant sources represent one of the most chemically diverse classes of small‐molecule therapeutics known to date. This remarkable diversity has long positioned medicinal plants as a cornerstone of traditional healing systems and, more recently, as a vital resource for modern drug discovery. Of approximately 374,000 known plant species worldwide, nearly 9% have been used for medicinal purposes (Woo et al. 2023), underscoring the immense and largely untapped therapeutic potential of botanical resources. Consequently, medicinal plants continue to offer a promising platform for the discovery of alternative antimicrobial and anticancer agents (Chaachouay and Zidane 2024).

Within this vast phytochemical landscape, plants belonging to the genus *Piper* (family Piperaceae) have received considerable scientific attention due to their extensive traditional use and broad pharmacological activities. *Piper chaba* Hunter, locally known as *Chui Jhal*, is an economically important edible flowering vine widely distributed throughout the warmer regions of Asia, including Bangladesh (Islam et al. 2020). Traditionally, *P. chaba* has been used to manage diarrhea, rheumatic pain, gastralgia, dyspepsia, piles, asthma, and other ailments, reflecting its long‐standing role in ethnomedicine. In recent years, phytochemicals isolated from various *Piper* species have demonstrated significant antimicrobial and anticancer activities, highlighting their relevance in contemporary therapeutic research (Salehi et al. 2019).

The growing interest in *Piper* species is closely linked to the escalating global burden of antimicrobial resistance and cancer—two of the most formidable health challenges of the 21st century (Periferakis et al. 2025; Wiraswati et al. 2025). The rapid emergence of multidrug‐resistant pathogens, together with the rising incidence of malignancies worldwide, has intensified the demand for novel, safe, and effective therapeutic agents. Notably, approximately half of all drugs approved by the US Food and Drug Administration between 1981 and 2019 were natural products, their derivatives, or mimetics, a proportion nearly double that of purely synthetic drugs (Newman and Cragg 2020). Furthermore, more than 75% of FDA‐approved anticancer drugs are either natural compounds or derived from natural scaffolds (Zaimy et al. 2017), reinforcing the enduring importance of nature‐based drug discovery.

Globally, at least 10 species of *Piper* have been reported to possess anticancer properties (Wang et al. 2014). Recent studies have shown that green‐synthesized silver nanoparticles prepared using extracts of *Piper betel* and *Piper sarmentosum* exhibit significantly enhanced antibacterial activity against *Escherichia coli* compared to their corresponding crude extracts (Nguyen et al. 2021). Supporting these findings, Rajia et al. (2025) demonstrated that *P. chaba* and its silver nanoparticles exhibit synergistic antibacterial activity when combined with antibiotics against multidrug‐resistant uropathogenic bacteria (Rajia et al. 2025). Additionally, several *Piper* species—including *Piper longum* L., *Piper boehmeriifolium* Wall., *Piper sylvaticum* Roxb., *Piper cubeba* L., and *Piper nigrum* —have been traditionally employed in the treatment of abdominal tumors in different regions of the world (Holdsworth 2008; Soladoye et al. 2010; Chaveerach et al. 2008; Chen 2008; Bezerra et al. 2013) which was also observed to have a correlation with lung cancer hyperglycemia (Popevic et al. 2025).

Among the bioactive constituents of the Piperaceae family, piperine has gained considerable attention for its multifaceted anticancer activity. Both piperine and its nanoformulations induce cell cycle arrest and apoptosis by regulating cyclins, cyclin‐dependent kinases (CDKs), and Bax/Bcl‐2 signaling pathways, and they also demonstrate synergistic effects with conventional chemotherapeutic agents (Ghasemi et al. 2025). At the molecular level, mutations in the tumor suppressor p53—the “guardian of the genome”—occur in nearly 50% of human cancers, resulting in dysregulated cell proliferation, while cyclooxygenase‐2 (COX‐2) is frequently overexpressed in several malignancies and promotes tumor growth, angiogenesis, invasion, and metastasis. Molecular docking studies using AutoDock Vina revealed that piperine binds strongly to p53 (−8.72 kcal/mol), with its 1,3‐dioxolane methoxy group forming a key hydrogen bond with the ILE100 residue (Dey et al. 2024). Molecular docking analysis of COX‐2 with compounds from *Piper longum* was performed in 2021 (Tripathi et al. 2021). More recently, It became evident that piperine from *Piper nigram* produced a more stable complex with COX‐2 than its native ligand arachidonic acid. Represented by their lower ΔGbind values (Gueroui et al. 2025). These findings highlight the therapeutic potential of Piper phytochemicals and provide a strong rationale for exploring their interactions with p53 and COX‐2 to support the rational design of plant‐based antimicrobial and anticancer agents.

This study investigates the antimicrobial and anticancer potential of *P. chaba* root extracts against Ehrlich ascites carcinoma (EAC) cells. Additionally, it focuses on the green synthesis of silver nanoparticles using *P. chaba* root extracts and evaluates their antimicrobial, cytotoxic, and anticancer properties. Moreover, this study employs computational biology to assess the binding affinity of *P. chaba* bioactive compounds with key cancer‐related protein ligands, providing insights into their therapeutic potential. An in silico drug design analysis was also conducted to explore the feasibility of developing novel anticancer agents.

## 2 Materials and Methods

### 2.1 Molecular Docking Analysis

Bioactive ligands were retrieved from the PubChem database in 3D SDF format and converted to PDB format using PyMOL. Ligand preparation was performed in AutoDock Tools prior to docking. Three‐dimensional structures of cancer cell target proteins were obtained from the RCSB PDB, and active binding sites were predicted using BIOVIA Discovery Studio. Molecular docking of selected phytocompounds was carried out using AutoDock Vina 4.0 following standard protocols (under default comprehensiveness parameters), and binding affinities were evaluated. The box size and grid box center were set for p53 (PDB ID: 4D1L) at X: 9.5213 Å, Y: 12.4132 Å, and Z: 68.3132 Å with a box size of X: 24.4345 × Y: 28.3733 × Z: 22.2334 Å. For COX‐2 (PDB ID: 1CX2), the grid center was at X: 23.4123 Å, Y: 22.5823 Å, and Z: 29.7417 Å with a box size of X: 26.8752 × Y: 26.3432 × Z: 28.3433 Å. These settings ensured proper coverage of the active binding regions during docking. Docking validation was performed through re‐docking of ligands, and docking reliability was evaluated by RMSD values < 2.0 Å. Protein–ligand interactions and docked conformations were analyzed using BIOVIA Discovery Studio. The top‐scoring complex was further subjected to molecular dynamics simulation using Desmond (Schrödinger v3.8), and ligand–protein interaction stability was assessed using the Desmond simulation interaction analysis tools.

### 2.2 Preparation of Root Extracts and Synthesis of Silver Nanoparticles From *P. chaba*

*Piper chaba* roots were collected from a local market in Khulna, Bangladesh, and taxonomically authenticated by the Department of Botany, University of Rajshahi, Bangladesh. The roots were washed with distilled water, cut into small pieces, sun‐dried, and ground into powder. A total of 250 g powder was extracted separately with distilled water and ethanol (1500 mL each) by continuous stirring for 72 h. The extracts were filtered, concentrated using a rotary evaporator at 45°C, and stored at 4°C until further use. Silver nanoparticles from *P. chaba* were synthesized following the procedure of our previous study (Rajia et al. 2025).

### 2.3 Determination of Antibacterial Activity

The antimicrobial activity of PcRE (Aq), PcRE (Et), and PcRE (Np) was evaluated using the standard broth microdilution method in 96‐well microtiter plates (Ameh et al. 2023). Growth inhibition was assessed against *Bacillus cereus* (ATCC 14579) and *E. coli* (ATCC 27853) at varying extract concentrations. Overnight bacterial cultures were adjusted to approximately 1 × 10^8^ CFU/mL prior to inoculation. Fresh nutrient broth without bacterial inoculum served as the blank, while inoculated broth without extract was used as the control. Following incubation at 37°C for 24 and 48 h, bacterial growth was quantified by measuring absorbance at 630 nm, and percentage growth inhibition was calculated relative to the control.

### 2.4 Determination of Antibiofilm Activity

The antibiofilm activity of PcRE (Aq), PcRE (Et), and PcRE (Np)—was evaluated using the crystal violet microtiter plate assay (Arfin et al. 2022). Briefly, 170 μL of an overnight culture of biofilm‐forming *E. coli* (ATCC 27853) (≈10^6^ CFU/mL) was inoculated into 96‐well flat‐bottom microtiter plates containing nutrient broth and adjusted to an optical density of 1.0 at 630 nm. Subsequently, 30 μL of serially diluted PcRE (Aq), PcRE (Et), and PcRE (Np) was added to obtain final concentrations of 31.25, 62.5, 250, 500, 1000, and 1500 μg/mL. Wells containing bacterial suspension without extract served as the negative controls. Azythromycin (10 mg/mL), an antibiotic, was used as the positive control. Plates were incubated at 37°C for 48 h to allow biofilm formation.

After incubation, planktonic cells were carefully removed, and each well was stained with 20 μL of 0.1% crystal violet (filtered through a 0.45 μm membrane) for 10 min at room temperature. Excess stain was discarded, and the wells were washed three times with phosphate‐buffered saline (PBS, pH 7.5) to remove unbound dye. The adhered biofilm was air‐dried, and 150 μL of 95% isopropanol was added to solubilize the bound crystal violet. After 15 min, absorbance was measured at 570 nm using a microtiter plate reader. Biofilm inhibition was expressed as percentage reduction in absorbance relative to the control using the following equation:

$$\%\text{Biofilm reduction}=\left({\mathrm{OD}}_{\text{control}}–{\mathrm{OD}}_{\text{treated}}\right)/{\mathrm{OD}}_{\text{control}}$$

In another experiment, bacterial biofilms were generated using the standard static microtiter plate and were subjected to fluorescence microscopy. Briefly, an overnight culture of *E. coli* (ATCC 27853) was established and subsequently inoculated into fresh medium containing either the target protein (treatment group) or no protein (control group). Following an additional 24 h incubation, bacterial smears were prepared and stained with 4′,6‐diamidino‐2‐phenylindole (DAPI; 1 μg/mL) for 10 min in the dark. Fluorescence images were then acquired using a microscope equipped with a UV filter (excitation/emission: 358/461 nm), and biofilm‐associated biomass was evaluated by comparing fluorescence intensity and coverage between treated and control samples.

### 2.5 Cytotoxicity Test by Brine Shrimp Nauplii Lethality Bioassay

Using the method described by Finney DJ, the cytotoxic activity of *P. chaba* extracts was evaluated by the brine shrimp ( *Artemia salina* L.) lethality bioassay. Artificial seawater was prepared, and actively swimming nauplii were collected after 24 h of hatching (Finney 1971). Ten nauplii were transferred into each vial containing artificial seawater, followed by the addition of graded concentrations of PcRE (Aq) and PcRE (Et) (37.5–500.0 μg/mL) and PcRE‐Np (37.5–250.0 μg/mL). Control vials contained no extract. All treatments were performed in triplicate and maintained at room temperature. After 24 h of exposure, the number of dead nauplii was recorded. The median lethal concentration (LC_50_) was calculated by plotting percentage mortality against extract concentration using probit regression analysis.

### 2.6 Determination of In Vivo Anticancer Potential of PcRE Extracts and Synthesized Nanoparticles

#### 2.6.1 In Vivo Anticancer Evaluation Using EAC Cells From Swiss Albino Mice

Healthy female Swiss albino mice (25–35 g, 6–8 weeks old) were obtained from the Department of Zoology, University of Rajshahi, Bangladesh, and housed under standard laboratory conditions (12 h light/dark cycle, 22°C ± 2°C) with free access to pellet diet and water. EAC cells were adjusted to a concentration of 4 × 10^6^ cells/mL, and 0.1 mL of the viable cell suspension was injected intraperitoneally into four groups of mice (*n* = 6 per group) to induce ascites tumors. After 24 h of tumor inoculation, one group of mice was kept as the ‘Control’ group and the other three groups received 2.5 mg/kg/day of PcRE (Aq), PcRE (Et) or PcRE (Np) for seven consecutive days (Bari et al. 2021) From the mice, ascetic fluid was collected and the tumor cells were extracted in sterile 0.9% sodium chloride saline and the number of viable EAC cells was counted with a hemocytometer by the trypan blue dye exclusion assay. We used the following formula to determine the percentage of tumor growth inhibition:

$$\text{Growth inhibition}\;\left(\%\right)=100-\left(\text{viable cells in treated group}/\text{viable cells in control group}\right)\times 100$$

#### 2.6.2 Determination of the Survival Rate of EAC‐Bearing Mice

The mortality of all EAC‐bearing control and treated mice groups was recorded daily, and the percentage increase in their life span (ILS) was calculated as follows:

$$\%\mathrm{ILS}=\left\{\left(T–C\right)/C\right\}\times 100$$

where *T* = mean survival time (or median survival time) of the treated group, *C* = mean survival time (or median survival time) of the control group.

#### 2.6.3 Determination of Hematological Parameters

On Day 8, all mice were sacrificed, and blood was collected in heparinized tubes. Red and white blood cells were counted using a hemocytometer, and hemoglobin levels were estimated (Aldayel et al. 2023). Percent reduction relative to the control was calculated as follows:

$$\%\text{Inhibition}=\left\{\left(A-B\right)/A\right\}\times 100$$

where *A* = Cells from control mice, *B* = Cells from PcRE (Aq), PcRE (Et) and PcRE (Np)‐treated mice.

#### 2.6.4 Fluorescent Microscopic Observation of EAC Cells From *P. chaba* Extract and Nanoparticle‐Treated Mice

EAC cells treated with PcRE (Aq), PcRE (Et), or PcRE (Np) samples were collected from the mice and washed thrice with phosphate‐buffered saline (PBS). After staining with Hoechst 33342 dye (0.1 μg/mL) at 37°C for 20 min, the unbound dye was removed by washing with PBS. Then these cells were subjected to a fluorescence microscope and observed for possible apoptotic changes (Islam et al. 2024).

### 2.7 MTT Assay‐Based In Vitro Assessment of the Anticancer Potential of PcRE Extracts and Nanoformulations

EAC cells were collected from tumor‐bearing Swiss albino mice following standard procedures (Islam et al. 2024). Viable cells (2 × 10^4^ in 100 μL) were seeded in 96‐well flat‐bottom plates containing DMEM and incubated at 37°C for 24 h. Various concentrations of PcRE‐Aq, PcRE‐Et, and PcRE‐Np were added to the wells, with three untreated wells serving as controls. After incubation, the media was replaced with 10 mM PBS, and 200 μL of MTT solution (5 mg/mL in PBS) was added, followed by incubation for 6 h at 37°C. The reagent was removed, and 200 μL of acidic isopropanol was added to solubilize the formazan crystals. Absorbance was measured at 570 nm, and cell growth inhibition (%) was calculated as follows:

$$\%\text{Inhibition}=\left\{\left(A–B\right)/A\right\}\times 100$$

where *A* = OD_570nm_ of the cellular homogenate from control well, *B* = OD_570nm_ of the cellular homogenate from treated well with PcRE (Aq), PcRE (Et), and PcRE (Np).

### 2.8 Determination of the Antidiabetic Activity of PcRE Extracts and Synthesized Nanoparticles

After 7 days of treatment, fasting blood glucose levels in tumor‐bearing mice were measured using a OneTouch glucometer (Glucox TD‐4183 blood glucose test strips, Germany) based on the glucose oxidase–peroxidase method.

### 2.9 Histopathological Examination of the Liver Sections of EAC‐Bearing Control and Sample‐Treated Mice

Cancer tissue samples were staged, graded, and typed using histopathology and immunohistochemistry techniques. Histological analysis of tumor and adjacent healthy tissues was performed following previously established protocols to evaluate tissue architecture and pathological changes (Parial et al. 2025).

### 2.10 Statistical Analysis

Data are presented as mean ± SEM from three independent experiments and analyzed using one‐way ANOVA followed by Dunnett's test (SPSS v21). Significance was indicated as *p < 0.05, **p < 0.01, ***p < 0.001.

## 3 Results

### 3.1 Molecular Docking Analysis

Molecular docking was conducted to assess the binding affinities of 11 bioactive compounds from *P. chaba* against tumor suppressor protein cyclooxygenase‐2 (COX‐2) and p53. Piperine exhibited the strongest interaction with COX‐2 (−8.9 kcal/mol), demonstrated stable interactions with key amino acid residues (CIYS: 36, CYS: 47, PRO: 153, GLU: 465, ARG: 44, ARG: 469, and GLN: 461) within the active binding pocket through hydrophobic (6) contacts and hydrogen (1) bonding, potentially contributing to enhanced binding stability (Figure 1A–C). Followed by (E)‐9‐(Benzo[d][1,3]dioxol‐5‐yl)‐1‐(piperidine‐1‐yl)non‐8‐en‐1‐one (−7.8 kcal/mol) and Myristicin (−6.7 kcal/mol) (Figure 1D–F,G–I), respectively. Similarly, against protein p53, Piperine (−6.9 kcal/mol) interacted with amino residues (ALA: 333, ALA: 336, THR: 306, GLU: 326, ARG: 310 and ARG: 340) implicated in catalytic activity through hydrophobic (5) interactions and hydrophilic (1) interactions, supporting its inhibitory potential (Figure 2A–C). Followed by (E)‐9‐(Benzo[d][1,3]dioxol‐5‐yl)‐1‐(piperidine‐1‐yl)non‐8‐en‐1‐one (−6.7 kcal/mol) and Myristicin (−5.9 kcal/mol) (Figure 2D–F,G–I), respectively. These results indicate their potential therapeutic relevance in modulating COX‐2 and p53 activity. Table 1 shows the molecular docking scores of some bioactive compounds for COX‐2 and p53 proteins.

**FIGURE 1:**
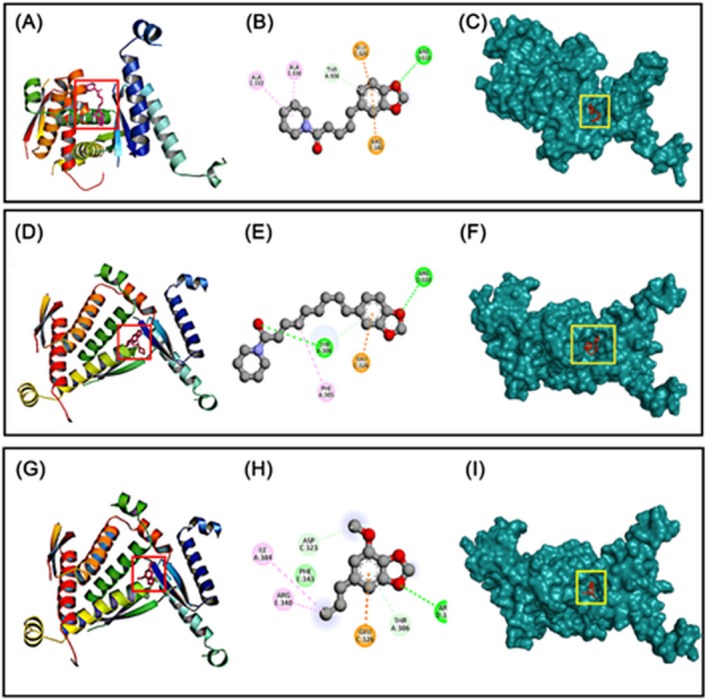
Molecular docking of *Piper chaba* compounds with COX‐2 protein (PDB ID: 1CX2). Interactions of CID‐638024 (A–C), CID‐21580213 (D–F), and CID‐4276 (G–I) are shown in cartoon, 2D, and surface views, respectively, illustrating binding interactions and conformations within the COX‐2 active sites.

**FIGURE 2:**
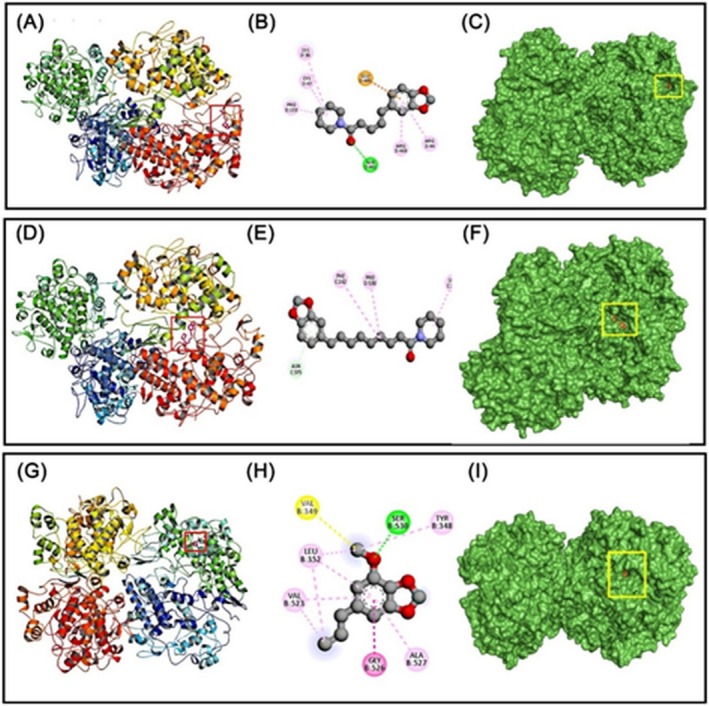
Molecular docking of *Piper chaba com*pounds with p53 (PDB ID: 4D1L). CID‐638024 (A–C), CID‐21580213 (D–F), and CID‐4276 (G–I) are shown in cartoon, 2D, and surface views, respectively, illustrating binding interactions and conformations within the p53 active sites.

**TABLE 1:** Molecular docking scores (binding affinities, kcal/mol) of selected bioactive compounds with p53 and COX‐2 proteins.

Compound	p53 (kcal/mol)	COX‐2 (kcal/mol)
Piperine	−6.9	−8.9
Piperitone	−5.7	−5.7
3‐Carene	−4.7	−5.9
Butane, 2‐(2,2‐dichloro‐1,3‐dimethylcyclopropyl)	−4.4	−5.1
8‐Heptadecene	−4.2	−5
d‐Limonene	−5.1	−6.3
Memantine	−5.2	−6.5
Myristicin	−5.9	−6.7
(E)‐9‐(Benzo[d][1, 3] dioxol‐5‐yl)‐1‐(piperidine‐1‐yl)non‐8‐en‐1‐one	−6.7	−7.8
n‐Hexadecanoic acid	−4.4	−5.3
α‐Pinene	−4.7	−5.7

*Note:* More negative values indicate stronger predicted binding affinity of the compound toward the target protein.

### 3.2 Antibacterial Activity

Using the turbidity assay, the MIC and MBC values of PcRE (Aq), PcRE (Et), and PcRE (Np) were evaluated against *E. coli* (ATCC 27853) and *Bacillus cereus* (ATCC 14579). All extracts exhibited stronger antibacterial activity against *E. coli* , reflected by comparatively lower MIC and MBC values (Table 2). Notably, PcRE (Np) showed the highest antibacterial potency against both strains, with the lowest MIC and MBC values. The MBC/MIC ratios (≤ 4) confirmed the bactericidal nature of all tested extracts. The superior efficacy of PcRE (Np) is likely due to enhanced surface area and improved bioavailability of bioactive phytoconstituents associated with nanoparticle formulation.

**TABLE 2:** Minimum inhibitory concentrations (MIC) and minimum bactericidal concentrations (MBC) of PcRE (Aq), PcRE (Et), and PcRE (Np) against Gram‐negative and Gram‐positive bacteria.

Extracts	Bacteria (−ve)	MIC (μg/mL)	MBC (μg/mL)	MBC/MIC ratio
PcRE (aq)	*E. coli* (ATCC 27853)	250	500	2.0
PcRE (Et)		230	500	2.2
PcRE (Np)		125	330	2.64

Extracts	Bacteria (+ve)	MIC (μg/mL)	MBC (μg/mL)	MBC/MIC ratio
PcRE (aq)	*Bacillus cereus* (ATCC 14579)	300	640	2.1
PcRE (Et)		290	640	2.06
PcRE (Np)		250	400	1.6

### 3.3 Antibiofilm Activity

The antibiofilm activity of PcRE (Aq), PcRE (Et), and PcRE (Np) was assessed at concentrations ranging from 31.25 to 1500 μg/mL. All extracts showed a dose‐dependent inhibition of *E. coli* (ATCC 27853) biofilm formation. At 31.25 μg/mL, absorbance values were 1.3, 0.95, and 0.846 for PcRE (Aq), PcRE (Et), and PcRE (Np) respectively. Increasing concentrations progressively reduced biofilm formation, with values of 0.264, 0.221, and 0.16 at 1000 μg/mL, and 0.14, 0.119, and 0.09 at 1500 μg/mL (Figure 3). Among the extracts, PcRE (Np) exhibited the most potent antibiofilm activity, indicating enhanced efficacy in nanoparticle form.

**FIGURE 3:**
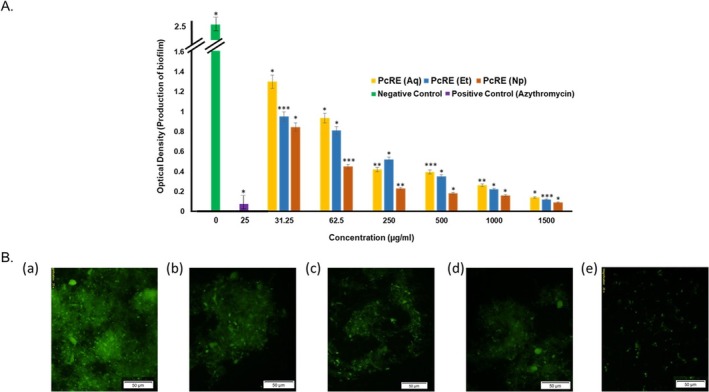
Antibiofilm activity of *PcRE* at varying concentrations against *Escherichia coli* (ATCC 27853). (A) Concentrations tested were 31.25, 62.5, 250, 500, 1000, and 1500 μg/mL (indicated as 1–6) respectively. The negative control contained only bacteria whereas the positive control was an antibiotic azithromycin (10 mg/mL). (B) Fluorescence view of biofilm‐producing bacteria after DAPI‐staining. (a) Negative control (only bacteria), (b) PcRE (Et)‐treated bacteria, (c) PcRE (Aq)‐treated bacteria, (d) PcRE (Np)‐treated bacteria and (e) azithromycin‐treated bacteria. Data are presented as mean absorbance values ± standard deviation of three independent experiments.

### 3.4 Cytotoxicity of PcRE (Aq), PcRE (Et), and PcRE (Np) by Brine Shrimp Nauplii Lethality Assay

The brine shrimp lethality assay was employed as a preliminary indicator of cytotoxicity, as the pharmacological and anticancer potential of nanoparticle formulations is closely associated with their toxic effects on living cells. Upon exposure of brine shrimp nauplii to varying concentrations of *P. chaba* extracts, the PcRE (Np) exhibited an LC_50_ value of approximately 1306.98 μg/mL, indicating moderate toxicity. In comparison, the PcRE (Aq) and PcRE (Et) extracts showed LC_50_ values of 2445.56 μg/mL and 1983.56 μg/mL, respectively (Figure 4). These findings suggest concentration‐dependent cytotoxic effects, with variability in toxicity among the different extract formulations.

**FIGURE 4:**
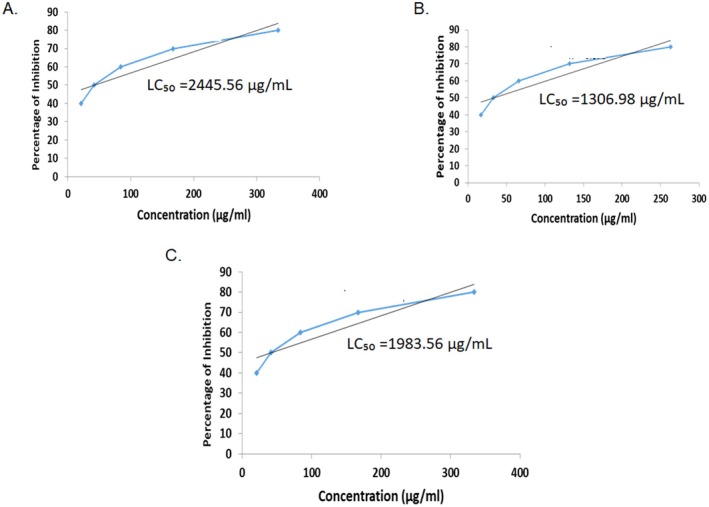
Cytotoxic activity of (A) PcRE (Aq), (B) PcRE (Np) and (C) PcRE (Et) in the brine shrimp lethality assay.

### 3.5 In Vivo Anticancer Activity of PcRE (Aq), PcRE (Et) and PcRE (Np) Against EAC Cells in Swiss Albino Mice

#### 3.5.1 In Vivo Cell Growth Inhibition of EAC‐Bearing Mice by PcRE (Aq), PcRE (Et) and PcRE (Np)

At the end of 7 days, the growth of EAC cells were inhibited by the treatment of PcRE (Et), PcRE (Aq) and PcRE (Np) with the comparing to the untreated or control cells. 26.3%, 32.0% and 41.5% of vivo cell growth inhibition was observed at 2.5 mg/kg/day doses of PcRE (Et), PcRE (aq) and PcRE (Np) respectively (Figure 5A).

**FIGURE 5:**
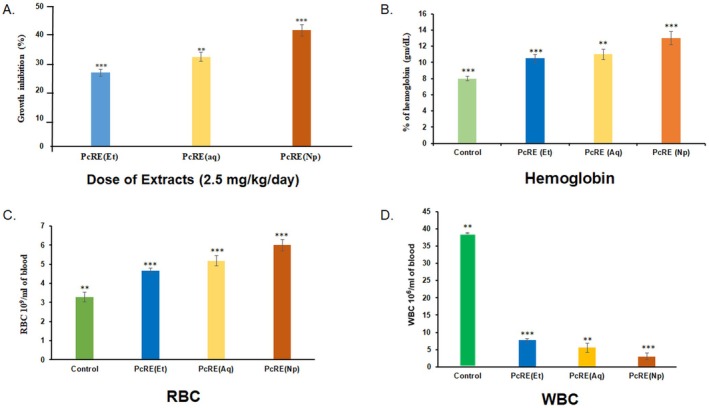
In vivo anticancer effects of PcRE extracts and nano formulations. (A) In vivo antiproliferative effects of PcRE on EAC cells compared to the untreated or control cells. (B) Hemoglobin concentration, (C) red blood cell (RBC) count, and (D) white blood cell (WBC) count, following treatment with PcRE (Et), PcRE (Aq), and PcRE (Np). Green bars represent the control group. Data are expressed as mean ± standard deviation (SD). Statistical significance was determined relative to the control group, where **p < 0.01, and ***p < 0.001.

#### 3.5.2 Hematological Parameters

Effects of PcRE (Et), PcRE (Aq), and PcRE (Np) on hematological parameters were evaluated in EAC‐bearing mice administered 2.5 mg/kg/day. RBC count, WBC count, and hemoglobin concentration were assessed (Figure 5B–D). All PcRE‐treated groups showed significant restoration of RBC and hemoglobin levels along with a marked reduction in WBC count compared to the control. The greatest decrease in WBC count was observed in the PcRE (Np) group (3.00 × 10^3^/μL), followed by PcRE (Aq) at 5.56 × 10^3^/μL and PcRE (Et) at 7.74 × 10^3^/μL, relative to the elevated control value 38.7 × 10^3^/μL. PcRE (Np) also produced the highest improvement in RBC count at 6.00 × 10^9^/mL and hemoglobin concentration 13.0 g/dL. These results suggest that PcRE, particularly PcRE (Np), alleviates EAC‐associated hematological abnormalities.

#### 3.5.3 Evaluation of Morphological Changes of PcRE (Aq) and PcRE (Np)‐Treated EAC Cells

Significant morphological changes were observed in the *P. chaba* root extract‐treated and synthesized nanoparticle‐treated EAC cells when compared to EAC cells from the control mice. Optical and fluorescent microscopy showed irregular‐shaped cells from the treated mice with reduced size (Figure 6B–F). These results suggest that PcRE and PcRE (Np) possibly induce apoptotic changes to the EAC cells in treated mice to exert anticancer effects.

**FIGURE 6:**
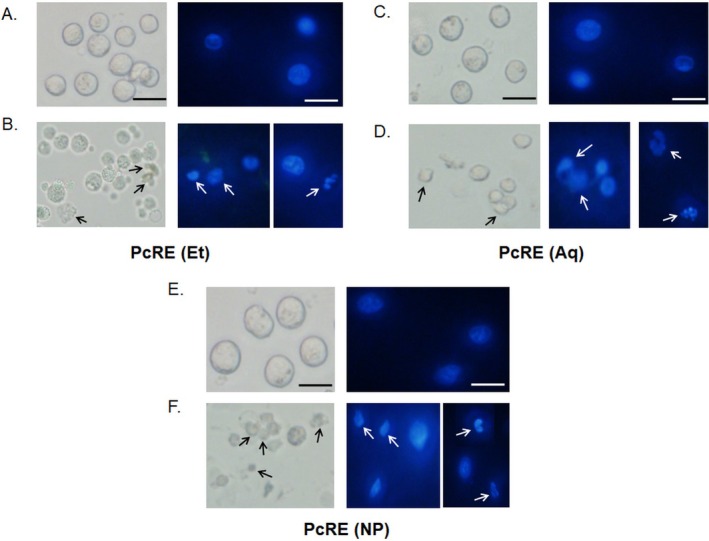
Morphological changes of PcRE extracts and Nano formulations‐treated EAC cells. (A), (C) and (E) showed optical and fluorescent views of control cells whereas (B), (D) and (F) represented PcRE (Et), PcRE (Aq) and PcRE (NP)‐treated EAC cells from Swiss albino mice, respectively. Scale bar: 25 μm.

### 3.6 In Vitro Antitumor Activity of PcRE (Aq) and PcRE (Np) Against EAC Cells Determined by MTT Assay

The antiproliferative effects of PcRE (Et), PcRE (Aq) and PcRE (Np) extracts on EAC cells were evaluated using the MTT assay. All extracts inhibited the cell growth in a dose‐dependent manner over the concentration range of 18.75–300 μg/mL. PcRE (Np) exhibited the strongest inhibition, with rates of 2.12%, 3.63%, 5.49%, 8.76%, and 12.7% at 18.75, 37.5, 75, 150, and 300 μg/mL, respectively. PcRE (Aq) and PcRE (Et) showed comparatively lower inhibition (0.54%–7.5% and 0.55%–6.65%, respectively). The IC_50_ values for PcRE (NP), PcRE (Et) and PcRE (Aq) were 1306.98, 1983.56 and 2445.56 μg/mL, respectively, indicating that the nanoparticle formulation mildly enhanced the antiproliferative activity in vitro (Figure 7A–C).

**FIGURE 7:**
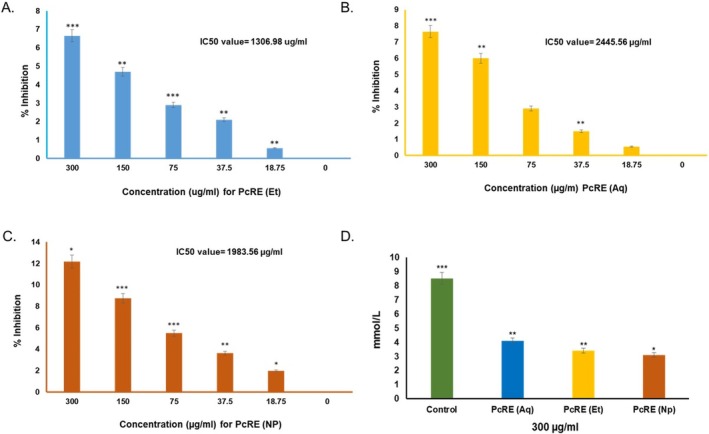
Dose‐dependent antiproliferative effects of *Piper chaba* extracts on EAC cells and their antidiabetic activity. (A) PcRE (Et); (B) PcRE (Aq); (C) PcRE (Np). Cells were treated for 48 h in DMEM medium. (D) Effect of PcRE extracts and nano formulations on fasting blood glucose level (BGL) in mice after 7 days of treatment. The control group showed elevated blood glucose levels, whereas PcRE‐treated groups exhibited a progressive reduction in blood glucose levels. Data are expressed as mean ± SEM. *p < 0.05, **p < 0.01, and ***p < 0.001.

### 3.7 Antidiabetic Activity of PcRE Extracts and Synthesized Nanoparticles

After 7 days of treatment, fasting blood glucose levels were the highest in the control group (8.5 mmol/L). Administration of PcRE (Aq), PcRE (Et), and PcRE (Np) markedly reduced blood glucose levels to 4.1, 3.4, and 3.1 mmol/L, respectively (Figure 7D). The glucose‐lowering effect followed a clear treatment‐dependent trend, with the most pronounced reduction observed in the PcRE (Np) treated group, indicating a strong antidiabetic potential of *P. chaba* root extracts.

### 3.8 The Survival Rate of EAC‐Bearing Mice

The results demonstrated that PcRE (Np) produced the greatest therapeutic effect, yielding a significant increase in life span of 52.60% in EAC‐bearing mice. PcRE (Aq) also showed appreciable activity, extending survival by 30.00%, whereas PcRE (Et) exhibited a comparatively lower effect of 18.66% (Figure 8A). These findings indicate that *P. chaba* extracts, particularly in nanoparticle form, contain bioactive constituents with the potential to enhance survival in EAC‐bearing mice.

**FIGURE 8:**
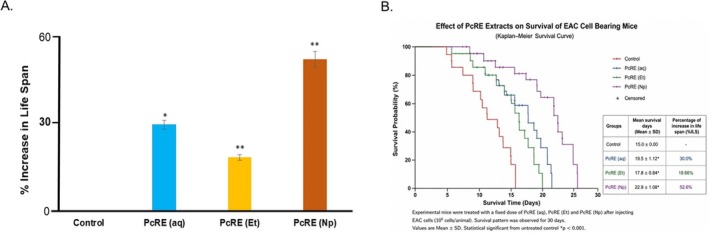
(A) Effect of PcRE (Aq), PcRE (Et) and PcRE (Np) extracts on the survival of EAC bearing mice (*n* = 6). Following intraperitoneal inoculation of EAC cells (1 × 10^6^ cells/animal), mice were treated with a fixed dose (2.5 mg/kg/day) of PcRE (Aq), PcRE (Et) and PcRE (Np). Survival was monitored for 7 days. (B) The Kaplan–Meier survival curve showed mean survival days and percentages of increase in life span for the control, PcRE (Aq), PcRE (Et), and PcRE (Np) groups. Data are expressed as mean ± SD. *p < 0.05, **p < 0.01, and ***p < 0.001.

### 3.9 Histopathological Examination of Liver Tissues in Control and PcRE (Aq) and PcRE (Np)‐Treated Mice

Histopathological examination of liver sections revealed distinct morphological variations among the experimental groups. In the control group, normal hepatic architecture with well‐organized hepatic lobules and a clearly defined central vein (C) was observed (Figure 9A). However, severe congestion of hepatic blood vessels accompanied by diffuse vacuolar degeneration of hepatocytes was found in the control group (Figure 9B). Liver tissues from the fluorouracil‐treated group exhibited pronounced treatment‐associated hepatic alterations, including disruption of hepatic cords, hepatocellular degeneration, and vascular congestion, indicating drug‐induced hepatic injury (Figure 9C). In contrast, the PcRE (Aq) treated group (Figure 9D) showed improved lobular organization with reduced cellular degeneration. Similarly, PcRE (Et) (Figure 9E) and PcRE (Np) (Figure 9F) treated groups exhibited comparatively normal hepatocytes and intact central veins. Overall, these findings indicate that PcRE treatments effectively mitigated fluorouracil‐associated hepatic alterations, suggesting a protective role of *P. chaba* root extracts in maintaining liver histoarchitecture during chemotherapy.

**FIGURE 9:**
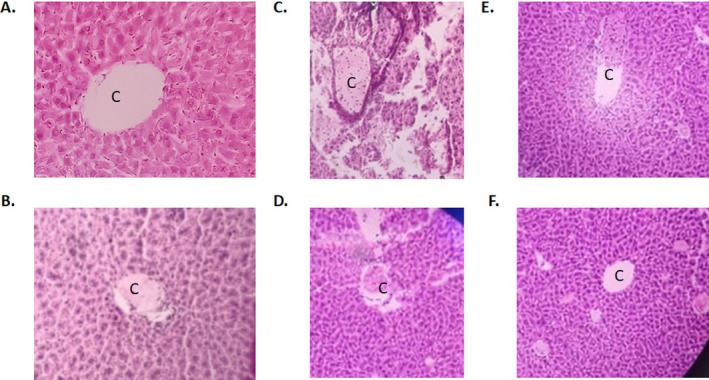
Histopathological evaluation of liver tissues in mice. (A) Normal hepatic architecture showing well‐arranged hepatic lobules, central vein ‘C’ and (B) Severe congestion of hepatic blood vessels and diffuse vacuolar degeneration of hepatocytes were observed in the control group (C) Liver sections from the fluorouracil‐treated group showing treatment‐associated hepatic changes (D–F) Liver tissues from mice treated with PcRE (Aq), PcRE (Et) and PcRE (Np), respectively, demonstrating preserved hepatic lobular structure with comparatively normal hepatocytes and central veins. (H&E staining, ×400).

## 4 Discussion

Synthesis and characterization of silver nanoparticles from *P. chaba* roots had already been performed by several research groups (Joshi Bachhar, Bhatia, et al. 2025; Joshi Bachhar, Mishra, et al. 2025), so the present study highlights the multifaceted biological activities of *P. chaba* root extracts (PcRE) and nanoparticle formulations PcRE (Np). Successful synthesis of PcRE‐derived silver nanoparticles (AgNPs) was evidenced by a distinct color change from transparent to dark brown, indicating the bioreduction of Ag^+^ to metallic Ag^0^, facilitated by phytochemicals acting as natural reducing and capping agents (Rajia et al. 2025). UV–visible spectroscopy further confirmed nanoparticle formation, revealing a characteristic surface plasmon resonance peak at 459 nm, consistent with previous reports (Parial et al. 2024; Almatroudi 2020).

Molecular docking studies provided mechanistic insights into the observed anticancer effects. Key bioactive phytochemicals, including piperine and (E)‐9‐(Benzo[d][1,3]dioxol‐5‐yl)‐1‐(piperidin‐1‐yl)non‐8‐en‐1‐one, demonstrated strong binding affinities toward cancer‐relevant targets such as p53 (Trp53) and COX‐2 (Table 1, Figures 1 and 2). Additional ligands, including D‐limonene, memantine, and myristicin, showed interactions with COX‐2‐related pathways, indicating potential multitargeted anticancer activity. These findings are consistent with previous studies reporting Piper‐derived compounds modulating VEGFR‐2, HER3, and NF‐κB signaling, leading to apoptosis and immunogenic cell death (Lister et al. 2020; Selvendiran et al. 2005; Duessel et al. 2008; Greenshields et al. 2015; de Souza Grinevicius et al. 2016; Park et al. 2020; Joshi et al. 2024; Joshi Bachhar, Mishra, et al. 2025).

PcRE exhibited significant antimicrobial activity against both Gram‐negative and Gram‐positive bacteria, with greater potency against *E. coli* (ATCC 27853). This differential susceptibility is likely attributable to variations in cell wall structure, as Gram‐negative bacteria possess an outer membrane that may enhance extract uptake in the presence of lipophilic phytochemicals. The antimicrobial efficacy of PcRE can be attributed to its rich phytochemical profile, including alkaloids, flavonoids, terpenoids, steroids, saponins, and tannins (Rahman et al. 2023; Al‐Mamun et al. 2024).

The antibiofilm assay further corroborated the antimicrobial potential of PcRE. PcRE (Aq), PcRE (Et), and PcRE (Np) formulations inhibited biofilm formation of *E. coli* (ATCC 27853) in a dose‐dependent manner across 31.25–1500 μg/mL. The PcRE (Np) exhibited the highest inhibition, likely due to enhanced penetration and interaction of bioactive constituents with bacterial biofilm matrices. These results align with prior reports on other Piper species, where solvent and formulation markedly influenced antibiofilm efficacy (Eawsakul et al. 2025; Everlyne et al. 2015; Kumar et al. 2013; Budzyńska et al. 2011).

Cytotoxicity evaluation using the brine shrimp lethality assay revealed that PcRE (Np) possessed higher cytotoxic potential than PcRE (Aq) and PcRE (Et), evidenced by lower IC_50_ values and greater inhibition at comparable concentrations. This enhancement can be attributed to increased bioavailability and cellular uptake afforded by the nanoparticle formulation. Comparatively, *Piper betle* and *Piper crocatum* exhibit higher cytotoxicity (LC_50_ ~31–45 μg/mL), whereas other plant‐mediated AgNPs, such as those from *Stereospermum kunthianum*, show higher LC_50_ values (~462 μg/mL) (Nerdy et al. 2021; Waghulde et al. 2019; Kabiru et al. 2022), suggesting that PcRE (Np) demonstrates moderate yet biologically relevant cytotoxicity with potential safety advantages.

In vivo studies using EAC‐bearing mice demonstrated that intraperitoneal administration of PcRE (2.5 mg/kg/day) significantly inhibited tumor growth. PcRE (Np) reduced EAC cell proliferation by 52.6%, exceeding the effects of PcRE (Aq) (30%) and PcRE (Et) (18.66%) (Figure 7), supporting the enhanced therapeutic efficacy of the nanoparticle formulation. Possible apoptotic changes had been observed in the EAC cells from extract‐ and nanoparticle‐treated mice. Hematological analysis revealed significant mitigation of tumor‐induced anemia. PcRE treatment restored red blood cell counts and hemoglobin levels while reducing elevated white blood cell counts, with PcRE (Np) exhibiting the strongest effect. These hematoprotective outcomes likely result from the extract's ability to modulate oxidative stress, reduce hemolysis, and normalize erythropoietin‐mediated erythropoiesis (Walker et al. 2009; Dhamija et al. 2013; Upadhyay et al. 2012; Patel et al. 2021; Morceau et al. 2009).

In a previous work, cancer‐induced control mice exhibited elevated fasting blood glucose levels, suggesting hyperglycemia and the involvement of shared molecular pathways linking cancer progression with altered glucose metabolism (Popevic et al. 2025). Joshi et al. found the antidiabetic potential of bimetallic silver‐iron nanoparticles using *P. chaba* extracts and determined their IC_50_ values to be 34.14 ± 0.73 μg/mL and 38.81 ± 0.65 μg/mL, respectively (Joshi Bachhar, Bhatia, et al. 2025). In this study, *P. chaba* root extracts, particularly the nanoparticle formulation [PcRE (Np)], significantly reduced fasting blood glucose from 8.5 mmol/L (control) to 3.1 mmol/L in cancer‐induced mice (Figure 8B). This marked reduction directly correlates with potential anticancer activity, as the treatment disrupts the metabolic demands of tumors by reversing cancer‐associated hyperglycemia. The study indicates that the superior hypoglycemic efficacy of the nanoparticles [PcRE (Np)] acts as a mechanism to starve glycolytically demanding cancer cells. Moreover, the reported hepatoprotective effects of red betel further corroborate the therapeutic potential of Piper species and support our observations shown in Figure 9 (Lister et al. 2020).

Taken together, the results demonstrate that PcRE, particularly in nanoparticle form, exhibits a broad spectrum of bioactivities, including antimicrobial, antibiofilm, cytotoxic, and antiproliferative effects. The enhanced efficacy of PcRE (Np) highlights the potential of nanoparticle formulations to improve bioavailability and potency of plant‐derived phytochemicals. Overall, these findings suggest that *P. chaba* root extracts represent promising multifunctional therapeutic agents with potential applications in antimicrobial, anticancer, and hematoprotective therapies.

## 5 Conclusion

This study demonstrates that PcRE, particularly in its silver nanoparticle formulation PcRE (NP), exhibits enhanced antimicrobial, antibiofilm, and anticancer activities. The green‐synthesized AgNPs showed superior bactericidal and antibiofilm efficacy, increased cytotoxicity, and significant inhibition of EAC growth, accompanied by prolonged survival and restoration of hematological parameters in tumor‐bearing mice. Molecular docking analysis further revealed strong interactions of major *P. chaba* phytochemicals, especially piperine with key cancer‐related targets, including p53 and COX2, providing mechanistic support for the observed bioactivities. Collectively, these integrated experimental and computational findings highlight PcRE derived silver nanoparticles as a promising multifunctional platform for the development of natural antimicrobial and anticancer therapeutics, warranting further pharmacokinetic and mechanistic investigations.

## Author Contributions

**Sultana Rajia:** conceptualization, methodology, data curation, formal analysis, visualization, writing – original draft, investigation. **Md. Ariful Islam:** methodology, formal analysis, investigation. **Md. Aminul Islam:** investigation, formal analysis, methodology. **Alima Khanam:** methodology, data curation, formal analysis, investigation. **Iffat Ara Tasnim:** methodology, formal analysis, investigation. **Md. Shamim Ahmed:** methodology, formal analysis, investigation. **Imtiaj Hasan:** conceptualization, funding acquisition, data curation, formal analysis, supervision, writing – review and editing, validation, resources.

## Funding

The research was funded by Varendra University Trust (VUT), Center for Interdisciplinary Research (CIR), Varendra University, Rajshahi, Bangladesh.

## Ethics Statement

Ethical approval of the experiments using Swiss albino mice was provided by the Ethical Review Committee (ERC) (Memo No. VU/ERC/2026/033), Varendra University, Rajshahi, Bangladesh.

## Conflicts of Interest

The authors declare no conflicts of interest.

## Data Availability

The data that support the findings of this study are available from the corresponding author upon reasonable request.
